# Cancer-associated fibroblasts induce epithelial–mesenchymal transition of bladder cancer cells through paracrine IL-6 signalling

**DOI:** 10.1186/s12885-019-5353-6

**Published:** 2019-02-11

**Authors:** Cassandra Ringuette Goulet, Audrey Champagne, Geneviève Bernard, Dominique Vandal, Stéphane Chabaud, Frédéric Pouliot, Stéphane Bolduc

**Affiliations:** 1 Centre de recherche en organogénèse expérimentale/LOEX, Regenerative Medicine Division, CHU de Québec-Université Laval Research Center, QC, Québec Canada; 2 Department of Surgery, Faculty of Medicine, Laval University, QC, Quebec Canada; 3 Oncology Division, CHU de Québec Research Center, QC, Quebec Canada; 4 Centre de recherche du CHU de Québec-Université Laval, Centre de recherche en organogénèse expérimentale de l’Université Laval/LOEX, 1401, 18e rue, Quebec city, Québec G1J 1Z4 Canada

**Keywords:** CAFs, IL-6, EMT, Bladder cancer

## Abstract

**Background:**

Cancer-associated fibroblasts (CAFs), activated by tumour cells, are the predominant type of stromal cells in cancer tissue and play an important role in interacting with neoplastic cells to promote cancer progression. Epithelial-mesenchymal transition (EMT) is a key feature of metastatic cells. However, the mechanism by which CAFs induce EMT program in bladder cancer cells remains unclear.

**Methods:**

To investigate the role of CAFs in bladder cancer progression, healthy primary bladder fibroblasts (HFs) were induced into CAFs (iCAFs) by bladder cancer-derived exosomes. Effect of conditioned medium from iCAFs (CM ^iCAF^) on EMT markers expression of non-invasive RT4 bladder cancer cell line was determined by qPCR and Western blot. IL6 expression in iCAFs was evaluated by ELISA and Western blot. RT4 cell proliferation, migration and invasion were assessed in CM ^iCAF^ +/− anti-IL6 neutralizing antibody using cyQUANT assay, scratch test and transwell chamber respectively. We investigated *IL6* expression relevance for bladder cancer progression by querying gene expression datasets of human bladder cancer specimens from TCGA and GEO genomic data platforms.

**Results:**

Cancer exosome-treated HFs showed CAFs characteristics with high expression levels of αSMA and FAP. We showed that the CM ^iCAF^ induces the upregulation of mesenchymal markers, such as N-cadherin and vimentin, while repressing epithelial markers E-cadherin and p-ß-catenin expression in non-invasive RT4 cells. Moreover, EMT transcription factors SNAIL1, TWIST1 and ZEB1 were upregulated in CM ^iCAF^-cultured RT4 cells compared to control. We also showed that the IL-6 cytokine was highly expressed by CAFs, and its receptor IL-6R was found on RT4 bladder cancer cells. The culture of RT4 bladder cancer cells with CM ^iCAF^ resulted in markedly promoted cell growth, migration and invasion. Importantly, inhibition of CAFs-secreted IL-6 by neutralizing antibody significantly reversed the IL-6-induced EMT phenotype, suggesting that this cytokine is necessary for CAF-induced EMT in the progression of human bladder cancer. Finally, we observed that *IL6* expression is up-regulated in aggressive bladder cancer and correlate with CAF marker *ACTA2*.

**Conclusions:**

We conclude that CAFs promote aggressive phenotypes of non-invasive bladder cancer cells through an EMT induced by the secretion of IL-6.

**Electronic supplementary material:**

The online version of this article (10.1186/s12885-019-5353-6) contains supplementary material, which is available to authorized users.

## Background

Bladder cancer is the 9th most commonly diagnosed cancer and is ranked 13th for cancer deaths in the overall population worldwide. Of all newly diagnosed cases, 75% present as non-muscle-invasive bladder cancer (NMIBC) disease, while 25% present as muscle-invasive bladder cancer (MIBC) disease with 10–15% of cases that are already metastatic [1, 2]. Of all solid cancers, bladder cancer has the highest rate of recurrence. After first line of treatment, 50 to 70% of patients with NMIBC will experience disease recurrence within 5 years and 10 to 30% of them will see their cancer progress to an invasive form [3–5].

The complex process of tumor metastasis consists of multiple steps during which cancer cells spread from primary tumor to other organs. Metastasis involves the epithelial–mesenchymal transition (EMT) process by which cancer cells transit between adherent epithelial and mobile mesenchymal states facilitating cancer cells dissemination. Epithelial cells loss E-cadherin expression, cell-cell adhesion and apico-basal polarization to gain vimentin expression and motility [6]. EMT is regulated by several transcription factors including snail homolog 1 (SNAIL1), twist basic helix–loop–helix transcription factor 1 (TWIST1) and zinc-finger E-box-binding homeobox 1 (ZEB1) [6]. Cadherin switch from E-cadherin to N-cadherin is a key step occurring during the EMT process [7]. This down-regulation of the E-cadherin and also the phosphorylation of GSK3β, an effector of Wnt signaling pathway, are associated with the release of β-catenin, which then translocates to the nucleus and activates the Wnt signaling pathway, known to be involved in metastasis formation. However, the precise molecular events that initiate this complex EMT process in bladder cancer are poorly understood. Increasing evidence suggests that the tumor microenvironment (TME) plays an important role in promoting EMT in tumor cells. Fibroblasts, the predominant stromal cell type in the TME, are activated by tumor cells into cancer-associated fibroblasts (CAFs) through the secretion of paracrine growth factors [8]. CAFs display a specific subset of markers, including α-smooth muscle actin (α-SMA; coded by *ACTA2* gene), fibroblast-activating protein (FAP), fibroblast-specific protein-1 (FSP1) and tenascin C [9, 10]. Previous studies suggest that CAFs play a pivotal role in establishing a metastatic niche and promoting tumor cell proliferation, invasion and metastasis by secretion of chemokines and cytokines in the microenvironment [9, 11, 12]. However, it is still unclear by which mechanisms CAFs affect the metastatic potential of bladder cancer cells.

IL-6 is a pleiotropic cytokine that modulates a variety of physiological events including metabolism, inflammation and immune response [13]. Activation of classic signalling requires binding of the IL-6 to its receptor (IL-6R) inducing the phosphorylation of signal transducer and activator of transcription 3 (STAT3), which dimerizes and translocates into the nucleus to regulate target gene transcription. A number of studies have highlighted the role of IL-6 and STAT3 in promoting tumor metastasis as their overexpression and/or hyper-activation have been reported in several human cancers [14–16]. Moreover, the level of IL-6 in blood of patients has been suggested as a prognostic marker [17]. Also, studies have shown that IL-6 contributes to cancer’s drug resistance [18]. IL-6 is overexpressed in bladder cancer tissues compared to non-malignant tissues at both mRNA and protein levels and elevated IL-6 levels correlated with higher clinical stage, higher recurrence rate after curative treatment, and reduced survival rate [19].

Although there is evidence suggesting that CAFs and IL-6 may be a critical factor in metastatic spreading, their role in EMT of bladder cancer cells remains unclear. Therefore, we designed this study to understand how CAFs may be promoting EMT in bladder cancer cells. Our results suggest that iCAFs induce EMT-related changes in cancer cells predominantly via the secretion of IL-6. We showed that the exposition of bladder cancer cells to the CAF conditioned medium (CM ^iCAF^) significantly induced the expression of N-cadherin, vimentin, SNAIL1, TWIST1 and ZEB1 while repressing E-cadherin and phospho-ß-catenin expression. In addition, the CM ^iCAF^ significantly enhanced cancer cell proliferation, migration and invasion. We also observed that *IL6* expression is up-regulated in aggressive bladder cancer tissues, correlates with CAF marker *ACTA2* and is associated with a poor prognosis. These results suggest an important role of IL-6 in mediating EMT and metastatic spreading of bladder cancer cells.

## Methods

### Ethics statement

Bladder biopsies from paediatric patients undergoing non-oncologic urologic surgery were obtained at the CHU de Québec Research Center in accordance with the institutional review board. All patients provided their formal, informed and written consent, each agreeing to supply a biopsy for this study.

### Cell isolation and culture

Healthy primary bladder fibroblasts (HFs) were isolated from two different human bladder biopsies as previously described [8, 20]. Briefly, the stroma was separated from the urothelium after incubation overnight at 4 °C in HEPES buffer with 500 μg/mL thermolysin (Sigma-Aldrich, Saint-Louis, MO). Fibroblasts were enzymatically dissociated from the extracellular matrix by treating the stroma with 0.125 U/mL collagenase H (Roche, Missisauga, Canada) for 3 h at 37 °C under gentle agitation. Then, fibroblasts were cultured in Dulbecco-Vogt modified Eagle’s media (DMEM) supplemented with 10% fetal bovine serum (FBS) (Invitrogen, Burlington, Canada) and antibiotics (100 U/ml penicillin and 25 μg/ml gentamicin; Sigma-Aldrich, Saint-Louis, MO). The results from the two HF populations were pooled together to minimize excessive data spread due to inter-individual variations. The RT4 bladder cancer cell line was obtained from the ATCC (HTB-2™) and cultured in DMEM containing 10% FBS and antibiotics. We selected the RT4 cells for our studies as a representative example of non-invasive bladder cancer cells [20]. Cells were cultured fewer than 5 passages after purchasing them for all the experiments and tested for mycoplasma contamination. Proteins were collected in RIPA buffer containing protease inhibitors COmplete (Roche, Missisauga, Canada), quantified using the BCA kit and store at − 80 °C until their use.

### Exosomes production and isolation

Exosomes were isolated as previously described [8]. Briefly, to remove any residual bovine exosomes from the FBS, serum was treated with the FBS Exosome Depletion Kit (Norgen Biotek Corp., ON, Canada) according to manufacturer’s instructions. Bladder cancer cells were cultured in DMEM containing 10% exosome-depleted FBS for 48 h. Then, the conditioned medium was centrifuged at 2000 g for 30 min to remove cells and debris, and the supernatant was mixed with 0.5 volume of the Total Exosomes Isolation Reagent (Invitrogen, Carlsbad, CA). Samples were mixed thoroughly by vortexing and incubated at 4 °C overnight. Then, they were centrifuged at 10,000 g for 60 min at 4 °C. Exosomes, contained in the pellet, were re-suspended in phosphate buffered saline (PBS). The protein concentration was measured by using the BCA kit (Pierce™, ThermoFisher, Waltham, MA).

### CAF induction

HFs were co-cultured with freshly isolated exosomes (1 mg/ml) for 48 h in DMEM 10% exosome-free FBS. Recombinant human transforming growth factor β1 (rhTGFβ1; 4 ng/mL, Peprotech, Rocky Hill, NJ) was used as a positive control. Then, the supernatant was aliquoted and store at − 80 °C until the ELISA assay, while proteins were collected in RIPA buffer containing protease inhibitors COmplete (Roche, Missisauga, Canada), quantified with the BCA kit and store at − 80 °C until the Western blots. RNAs were isolated using 1 mL of TRIzol Reagent (Invitrogen Corporation, Carlsbad, CA) and stored at − 80 °C until the RT-PCR assay.

### Preparation of the conditioned medium

HFs, HFs + TGFß or induced CAFs (iCAFs) were cultured in DMEM containing 10% FBS and antibiotics until they reached a confluency of approximately 80%, the cell culture medium was thus collected and centrifuged at 1200 g for 10 min. RT4 bladder cancer cells were cultured in freshly collected conditioned mediums (CM).

### Immunoblot

Equal amounts of proteins (10 μg) were loaded into 12% polyacrylamide gels, resolved by SDS-PAGE and transferred onto PVDF membranes. Membranes were blocked for 30 min with 5% non-fat milk and 0.05% Tween 20 in PBS, and then incubated with primary antibodies overnight at 4 °C followed by 45 min at RT with HRP-conjugated secondary antibodies (Jackson Immunoresearch Laboratories, West Grove, PA). The protein expression was detected using the Amersham ECL Prime Western Blotting Detection Reagent (GE Healthcare, Little Chalfont, UK). Bands were imaged by using the Fusion Fx7 imager (Vilber Lourmat, France) and analyzed with the ImageJ software (NIH, Bethesda, MD). The following antibodies were used: αSMA (1/5000; Abcam, San Francisco, CA), FAP (1/1000, Novus Biologicals, Littleton, CO), tubulin (1/1000; Novus Biologicals, Littleton, CO), E-cadherin (1/3000; R&D Systems, Minneapolis, MN), N-cadherin (1/3000; Millipore, Burlington, MA), vimentin (1/1000; Abcam, San Francisco, CA), phospho-β-Catenin (1/1000; Cell Signaling, Danvers, MA), total β-catenin (1/1000; Abcam, San Francisco, CA), pGSK3β (1/500; Cell Signaling, Danvers, MA), GSK3β (1/750; Cell Signaling, Danvers, MA), SNAIL1 (1/2000; Invitrogen, Carlsbad, CA), TWIST1 (1/1500; LifeSpan BioSciences, Seattle, WA), ZEB1 (1/1000; Abcam, San Francisco, CA), IL-6R (1/1000; Abcam, San Francisco, CA), pSTAT3 (1/1.000, Cell Signaling, Danvers, MA), STAT3 (1/1000; Cell Signaling, Danvers, MA) and β-actin (1/5000; Abcam, San Francisco, CA), pAKT (1/1000; Cell Signaling, Danvers, MA), AKT (1/1000; Cell Signaling, Danvers, MA).

### Elisa

IL-6 protein expression was quantified by using an ELISA according to the manufacturer’s protocol (Duoset; R&D Systems). The plates were read at 450 nm using a SpectraMax Plus spectrometer with SoftmaxPro Version 4.7.1.

### RT-qPCR

RNA was extracted using the TRIzol reagent according to the manufacturer’s instructions. RNA quality was assessed on a Bioanalyzer using the Agilent RNA 600 Nano Kit. The concentration and purity of the RNA was determined using a NanoDrop 2000 (ThermoFisher Scientific, Waltham, MA). One μg of RNA was used to reverse transcribed into cDNA using the High-Capacity cDNA Reverse Transcription Kit (Life Technologies, Carlsbad, CA). qPCR was performed in triplicate using the DyNAmo HS SYBR Green qPCR Kit (ThermoFisher Scientific, Waltham, MA) and Lightcycler® 480 system (Roche, Mississauga, Canada), following the manufacturer’s instructions. The β-2-microglobulin transcript was used as an endogenous control for normalization.

### Proliferation assay

RT4 cells were seeded in 96-well plates at a density of 3 × 10^3^ cells/well and allowed to attach for 12 h. Then, RT4 were treated with the CM from HFs, HFs + TGFß or iCAFs for 24 h in presence or absence of an anti-IL-6 antibody (1 μg/mL). Cell proliferation was assessed by fluorometric quantification of DNA using CyQUANT Proliferation Assay Kit (Life Technologies, Carlsbad, CA) according to the manufacturer’s instructions.

### Cell migration assay

RT4 cells were first seeded at a concentration of 4 × 10^4^ cells/100 μl in both chambers of an Ibidi-silicone insert (Ibidi, Martinsried, Germany). This insert allows for the formation of a well-defined edge without physically scratching or wounding the cell monolayer. Cells were cultured for 24 h in DMEM containing 10% FBS to form a confluent monolayer. Then, cells were grown in serum-free DMEM and Mitomycin C (5 μg/ml, Sigma #M4287) to inhibit cell proliferation for 12 h prior to careful removal of the insert. Cells were incubated in the CM from HFs, HFs + TGFß or iCAFs for 24 h in presence or absence of an anti-IL-6 antibody (1 μg/mL). The migration was visualized at the indicated times (0, 6, and 12 h) under an inverted microscope (TE2000, Nikon). Migration distances were measured using the ImageJ analysis software (National Institutes of Health, Bethesda, MD).

### Transwell invasion assays

Invasion assays were performed using transwell 24-well plates with 8-μm diameter filters (Corning, NY, USA). Filters were precoated with 40 μL of purified Type I bovine collagen gel (2.5 mg/mL, Sigma-Aldrich, Saint-Louis, MO) and incubated for 4 h at 37 °C. Approximately 1 × 10^5^ cells in 200 μL of serum-free DMEM + Mitomycin C (5 μg/ml) with or without an anti-IL-6 antibody (1 μg/ml) were placed in the upper chamber and 500 μL of the CM from HFs, HFs + TGFß or iCAFs was added in the lower chamber. The plates were incubated for 24 h. Then, cells on the upper side of the filters were removed with a cotton swab, and the filters were washed with PBS. Cells were fixed in methanol for 15 min and nuclei were stained with DAPI. The relative cell migration was determined by the number of migrated cells in 10 randomly selected fields.

### Mitomycin C sensitivity assay

RT4 cells were seeded in 96-well plates at a density of 3 × 10^3^ cells/well and allowed to attach for 12 h. Then, RT4 cells were treated with the CM from HFs, HFs + TGFß or iCAFs for 24 h in presence or absence of mitomycin C (0,5 μg/mL, Sigma-Aldrich, Saint-Louis, MO). Plates were washed with PBS to remove any debris and cell viability was assessed by fluorometric quantification of DNA using CyQUANT Proliferation Assay Kit (Life Technologies, Carlsbad, CA) according to the manufacturer’s instructions.

### Genomic data processing and tumor purity analysis

Clinicopathological profiles and genomic data from TCGA [21] and GSE13507 [22] were downloaded respectively on GDC (Genomic Data Common; https://portal.gdc.cancer.gov/) and GEO (Gene Expression Omnibus; https://www.ncbi.nlm.nih.gov/geo/) data portal (*n* = 412 and *n* = 272, respectively). The expression of *IL6* in human bladder cancer specimens was analyzed using GraphPad Prism 7.0 in bladder cancer patients linked with their clinical parameters and follow-up data information. Tumor purity of the TCGA datasets was obtained using the ABSOLUTE algorithm (https://confluence.broadinstitute.org/display/CGATools/ABSOLUTE (2013)). We applied a Pearson’s correlation to test for the association between tumor sample purity and mRNA expression of *IL6* and *ACTA2* expression.

### Statistical analysis

Graphical representation of data and statistical analysis was performed with the GraphPad Prism v.7 Software (San Diego, CA, USA). The results are expressed as mean ± standard error (SD) and were interpreted using one- or two-way analysis of variance (ANOVA). mRNA expression distribution was analyzed using the non-parametric Mann-Whitney U test. Survival analyses were determined by Kaplan-Meier method, where the difference was evaluated by the Log-rank test. Differences between the groups were considered significant at *P* < 0.05.

## Results

### Healthy bladder primary fibroblasts treated with bladder cancer-derived exosomes exhibit characteristics of CAFs

**Fig. 1:**
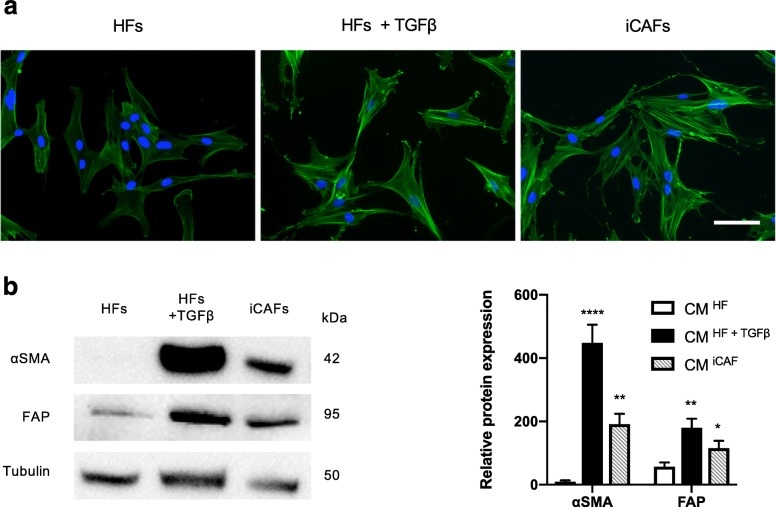
Healthy vesical primary fibroblasts (HFs) treated with bladder cancer-derived exosomes exhibit characteristics of cancer-associated fibroblasts (CAFs). HFs were treated with 1 mg/mL of bladder cancer cells-derived exosomes for 48 h to induce their activation in CAFs (iCAFs). HFs treated with TGFβ1 (4 ng/mL) served as a positive control. **a**. Cells were examined by immunocytochemistry for the expression of αSMA (α-smooth muscle actin, green). Nuclei were stained with DAPI (blue). Scale bar = 100 μm. **b**. The protein expression of αSMA, fibroblast-activating protein (FAP) was determined by western blot. The tubulin was used as loading control. The graph shows mean +/− SD. The difference between groups was analyzed by one-way ANOVA followed by post hoc analysis using Dunnett’s multiple comparison tests. *P < 0.05, **P < 0.01, ****P < 0.0001, *n* = 3

To induce the activation of healthy bladder primary fibroblasts (HFs) into CAFs (iCAFs), we cultured HFs with 1 mg/mL of bladder cancer-derived exosomes for 48 h [20]. HFs + TGFß was used as positive control for activated fibroblasts [8]. iCAFs were positive for αSMA staining and exhibited a spindle-shape morphology, similar to CAFs (Fig. 1a). Moreover, iCAFs presented higher levels of αSMA and FAP proteins expression (Fig. 1b).

### iCAFs induce EMT program in bladder cancer cells

**Fig. 2:**
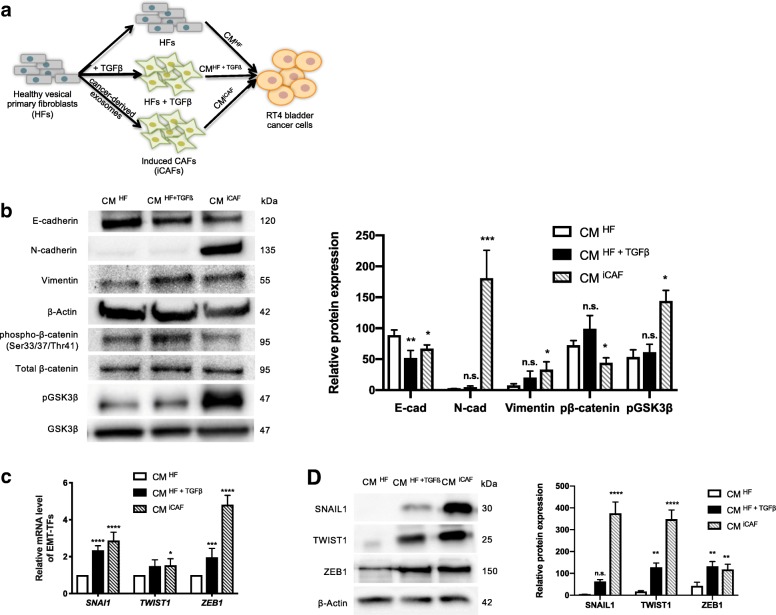
The conditioned medium (CM) from CAFs induces epithelial-mesenchymal transition (EMT) programming in RT4 bladder cancer cells. **a**. Experimental design. **b**. The expression of epithelial markers E-cadherin, phospho-ß-catenin (control: total ß-catenin) and phospho-GSK3ß (control: GSK3ß total) and of mesenchymal markers N-cadherin and vimentin was analyzed by Western blotting. The ß-actin was used as loading control. **c**. The expression of EMT-related transcription factors (EMT-TFs) was determined by qPCR or **d**. Western blotting. The graphs show mean +/− SD. The difference between groups was analyzed by one-way ANOVA followed by post hoc analysis using Dunnett’s multiple comparison tests. *P < 0.05, **P < 0.01, ***P < 0.001, ****P < 0.0001, n.s. = non-significant, *n* = 3–5

As EMT plays a pivotal role in tumor metastasis, we investigated the effects of iCAFs on cancer cells EMT, the CM ^iCAF^ was collected and used to grow the bladder cancer cell line RT4 (Fig. 2a). We examined changes of EMT phenotype induces by CM ^iCAF^ by measuring the protein expression of epithelial markers E-cadherin, phospho-ß-catenin and phospho-GSKß and of mesenchymal markers N-cadherin and vimentin. Results showed that RT4 cells cultured with CM ^iCAF^ had decreased expression of E-cadherin, phospho-ß-catenin and phospho-GSK3ß, while the expression of N-cadherin and vimentin was increased (Fig. 2b). To investigate whether the EMT programming was activated by the CAF secretome in RT4 cells, the expression of EMT-related transcription factors (EMT-TFs) SNAIL1, TWIST1 and ZEB1 was measured by qPCR. The results showed that *SNAI1* and *ZEB1* expression levels were highly upregulated, while *TWIST1* expression levels were slightly increased in RT4 cells cultured with CM ^iCAF^ (Fig. 2c). These results were also confirmed at protein level (Fig. 2d). These results suggested that the CAF secretome enhances the aggressive behaviour of bladder cancer cells by inducing an EMT phenotype through well-known EMT-TFs.

### iCAFs activate the STAT3 signalling pathway in bladder cancer cells via IL-6 secretion

**Fig. 3:**
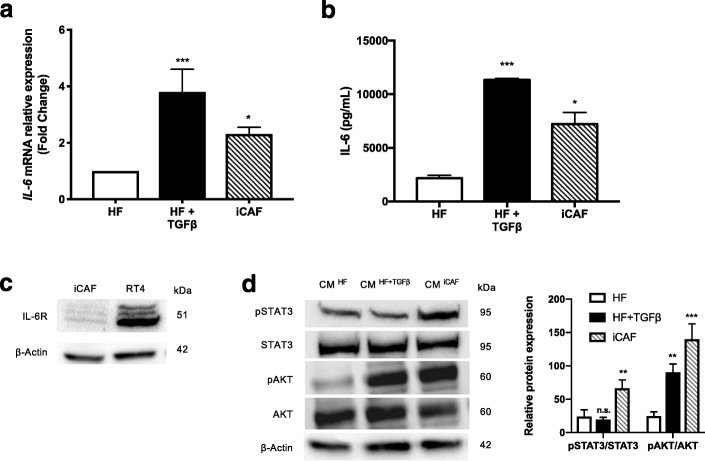
iCAFs express IL-6 and induce the activation of the STAT3 signaling pathway in RT4 bladder cancer cells. **a**. Interleukin-6 (IL-6) mRNA expression levels in HFs, HFs + TGFß and iCAFs cells were determined by qPCR. **b**. The expression of the IL-6 protein was measured in supernatants from HFs, HFs + TGFß and iCAFs cells using enzyme-linked immunosorbent assay (ELISA). **c**. The presence of the IL-6 receptor (IL-6R) in iCAFs and RT4 cells was detected by Western blotting. **d**. The activation of the signal transducer and activator of transcription 3 (STAT3) and AKT signaling pathway in RT4 cells cultured in conditionned medium (CM) was evaluated by Western blotting. The graphs show mean +/− SD. The difference between groups was analyzed by one-way ANOVA followed by post hoc analysis using Dunnett’s multiple comparison tests. *P < 0.05, **P < 0.01, ***P = 0.001, n.s. = non-significant, *n* = 3–5

The pro-inflammatory cytokine interleukin-6 (IL-6) has been shown as an important EMT inducer in breast and lung cancers [14, 15]. Therefore, we quantified the IL-6 mRNA expression in HFs, HFs + TGFß and iCAF cells as well as the IL-6 protein secretion in supernatants. Our results showed that iCAFs expressed more *IL6* mRNA and secreted significantly more IL-6 protein than HFs (Fig. 3a, b). In order to determine if RT4 cells could interact with iCAFs-secreted IL-6, we evaluated the expression of the IL-6R on RT4 cells. The IL-6R was highly expressed in RT4 cells compared to iCAFs, indicating that IL-6 could induce responses in RT4 cells (Fig. 3c). The canonical IL-6 signal transduction pathway is initiated by the cytokine binding to the IL-6R and the subsequent phosphorylation of STAT3. To investigate the effect of the CM ^iCAF^ on the activation of IL-6 signaling pathway in bladder cancer cells, we compared the expression levels of phosphorylated STAT3 (pSTAT3) and phosphorylated AKT (pAKT) in RT4 cells cultured with CM ^iCAF^ to control media (CM ^HF^ and CM ^HF + TGFß^). The results showed that CM ^iCAF^ significantly increased pSTAT3 and pAKT in RT4 cells compared to CM ^HF^, while total STAT3 and AKT expression remained unchanged (Fig. 3d).

### iCAFs-secreted IL-6 promotes the proliferation, migration and invasion of bladder cancer cells

**Fig. 4:**
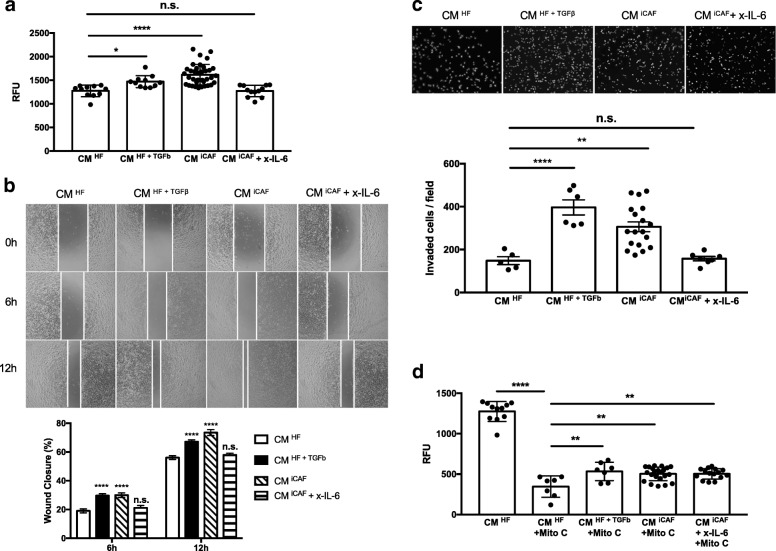
CAFs-derived IL-6 enhances survival and promotes migration and invasion in RT4 bladder cancer cells. RT4 cells were cultured in the CM from HFs, HFs + TGFß or iCAFs with or without the anti-IL-6 antibody (1 μg/mL; x-IL-6). **a**. The proliferation of the RT4 cells was evaluated using the CyQUANT Kit. **b**. The migration of RT4 cells was determined by using Ibidi-silicone insert. Insert margins were marked by white vertical lines on optical micrographs. After 6 h and 12 h, the percentage of wound closure was evaluated by measuring migration distances (spaces between the white vertical lines). **c**. The invasion of RT4 cells was measured by transwell assays. Cells that had migrated through the membrane were fixed and nuclei were stained with DAPI. Representative photographs of migratory cells on the membrane are shown. The relative cell migration was determined by the number of migrated cells in 10 randomly selected fields. **d**. The sensitivity of RT4 cells to mitomycin C (0.5 μg/mL; Mito C) was evaluated using The CyQUANT Kit. RFU means relative fluorescence unit. All values are averages of three independent replicates. The graphs show mean +/− SD. The difference between groups was analyzed by one-way ANOVA followed by post hoc analysis using Dunnett’s multiple comparison tests. *P < 0.05, **P < 0.01, ***P < 0.001, ****P < 0.0001, n.s. = non-significant, *n* = 5–25

The tumor metastatic cascade is characterized by the activation of EMT process in cancer cells, where loss of cell–cell junctions and cell polarity lead to the acquisition of migratory and invasive properties. Therefore, we investigated the role of iCAFs-secreted IL-6 on cell growth and motility of RT4 cells treated with CM ^iCAF^ with or without the anti-IL-6 neutralizing antibody. When RT4 cells were treated with CM ^iCAF^, the proliferation rate increased by 21.2% after 24 h (Fig. 4a). To test the specificity of the IL-6 stimulation, we added an anti-IL-6 antibody (x-IL-6) to the culture medium to block IL-6-induced proliferation. The activity of the IL-6 on cell proliferation of RT4 cells was reversed by the anti-IL-6 antibody (Fig. 4a). The migration assay was used to examine the effect of the iCAFs-secreted IL-6 on RT4 cells. As shown in Fig. 4b, the CM ^iCAF^ increased the migration activity of RT4 cells significantly. When RT4 cells were incubated with the CM ^iCAF^ and proliferation inhibitor, the migration rate after 6 h and 12 h was increased of 36.4 and 23.7%, respectively (Fig. 4b). To examine the effect of the iCAFs-secreted IL-6 on the invasion of RT4 cells, a transwell assay was used where artificial extracellular matrix (ECM) was precoated on the upper side of all filters. We found that the number of cells that invaded through the ECM was raised by 51.5% when RT4 cells were cultured in the CM ^iCAF^ compared to the CM ^HF^ (Fig. 4c). In addition, when RT4 cells were treated with the CM ^iCAF^ in combination with the anti-IL-6 antibody (1 μg/ml), the IL-6-induced cell migration and invasion was markedly inhibited (Fig. 4b, c). These data imply that the iCAFs-secreted IL-6 is clearly implicated in the proliferation, migration and invasion abilites of RT4 cells.

### The CM ^iCAF^ increases cancer cell survival in response to the chemotherapeutic drug mitomycin C

Chemotherapeutic agents such as mitomycin C have long been used to treat bladder tumors, but development of drug resistance remains a substantial problem [1, 23]. To investigate how CAFs might influence the sensitivity of bladder cancer cells to mitomycin C, RT4 cells were cultured in the CM from HFs, HFs + TGFß or iCAFs with 0.5 μg/mL mitomycin C. As expected, the viability of RT4 cells was significantly decreased by 72.9% after the addition of mitomycin C for 12 h, demonstrating the effectiveness of the drug (Fig. 4d). When cultured with the CM ^iCAF^, RT4 cells showed reduced cell death (12.3%) as compared with the CM ^HF^. However, cell death was not significantly reduced after the addition of the anti-IL-6 antibody to CM ^iCAF^ compared to the CM ^iCAF^ alone (12.4% vs 12.3%). Thus, cell death induced by mitomycin C was attenuated by the CM ^iCAF^ but was not mediated by the iCAFs-secreted IL-6.

### *IL6* expression is up-regulated in aggressive bladder cancer, correlates with the CAF marker *ACTA2* and stromal compartment and is associated with poor clinical outcome

**Fig. 5:**
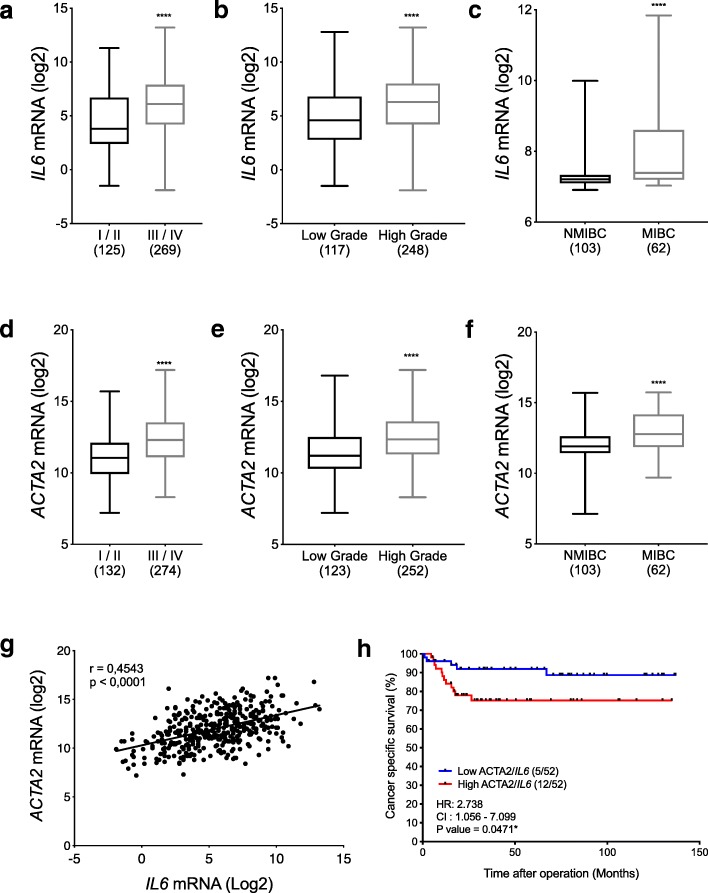
*IL6* mRNA expression is up-regulated in aggressive bladder cancer patient tumor specimens, correlates with CAF marker *ACTA2* expression, and is associated with poor prognosis. Boxplots of *IL6* (**a**-**c**) and *ATCA2* (**d**-**f**) mRNA expression with respect to tumor stage, grade or invasiveness for TCGA (**a**-**b**, **d**-**e**, **g**) and GSE13507 (**c**, **f**, **h**) bladder cancer data. Number of tissues in each group is shown in brackets. The difference between groups was analyzed by Mann-Whitney test. ****P < 0.0001. **g**. Spearman’s correlation between *ACTA2* and *IL6* gene expression (r = 0.4543; ****P < 0.0001). **h**. Kaplan-Meier cancer specific survival curves according to lower versus higher half of *ACTA2/IL6* mRNA co-expression level in bladder cancer patients (HR = 2.738; *P = 0.0471). Non-muscle invasive bladder cancer (NMIBC); muscle invasive bladder cancer (MIBC)

We analyzed *IL6* and *ACTA2* expression in the publicly available TCGA and GSE13507 gene expression profiling datasets of human bladder cancer samples [21, 22]. The stratification of patients according to tumor staging, grading and invasiveness revealed that *IL6* expression was found up-regulated in stage III/IV compared to stage I/II (****P < 0.0001), in high grade compared to low grade (****P < 0.0001) and in invasive compared to superficial bladder cancer (****P < 0.0001) (Fig. 5a-c). Similar results were obtained with CAF marker *ACTA2* expression, suggesting an activation of fibroblasts in aggressive bladder cancer (Fig. 5d-f). The expression levels of *IL6* and *ACTA2* were positively correlated (r = 0.4543; ****P < 0.0001; Fig. 5g). Moreover, Kaplan–Meier analysis of the cancer specific survival showed a significant correlation between patients with high versus low *IL6/ACTA2* expression (Fig. 5h, HR = 2.738, 95% CI = 1.056–7.099, *P = 0.0471). Tumor purity is the proportion of cancer cells within a tumor. Despite some selection for inclusion, TCGA tissue samples may retain a heterogeneous mix of cell types, causing varying levels of tumor purity. To independently assess the origin of *IL6* and *ACTA2* expression, we correlated bladder cancer tumor purity scores generated using the ABSOLUTE algorithm with the corresponding *IL6* and *ACTA2* expression. To this end, TCGA samples with matching RNAseq expression and ABSOLUTE-based tumor purity estimates (*N* = 399) were analyzed. We observed a highly negative correlation between the tumor purity and *IL6* mRNA (r = − 0.6274; ****P < 0.0001. Additional file 1) or *ACTC2* mRNA (r = − 0.4888; ****P < 0.0001. Additional file 2), indicating that higher average *IL6* and *ACTA2* expression correspond with higher stromal component of the tumor. We next investigated whether *ACTA2/IL6* mRNA co-expression in bladder cancer tumors correlated with clinicopathological features. For these analyses, bladder cancer patients were stratified based on low and high quartiles of *ACTA2/IL6* co-expression levels in tumors. As shown in Table 1, no significant association was observed between *ACTA2/IL6* mRNA co-expression and patient age, metastatic invasion, cancer recurrence or progression. A correlation was seen between high *ACTA2/IL6* co-expression and the tumor grade (*P = 0.0179) and lymph node metastasis (*P = 0.0281). A strong association was observed between high *ACTA2/IL6* mRNA co-expression and tumor invasiveness (****P < 0.0001), correlating with our in vitro findings.

**Table 1:** Correlation between clinicopathological features and ACTA2/IL6 mRNA co-expression in bladder cancer patients

Features	No. of patients	*ACTA2/IL6* expression		*p* value
		Low/Low	High/High	
Age (years)				
< 65	53	28 (52,8%)	25 (47,2%)	0.6950
> 65	51	24 (47,1%)	27 (52,9%)	
Gender				
Male	85	48 (56,5%)	37 (43,5%)	0.0058**
Female	19	4 (21,1%)	15 (78,9%)	
Invasiveness				
Superficial	67	43 (64,2%)	24 (35,8%)	0.0001****
Invasive	37	9 (24,3%)	28 (75,7%)	
Grade				
Low	75	43 (57,3%)	32 (42,7%)	0.0179*
High	29	9 (31,0%)	20 (69,0%)	
Lymph node metastasis				
N0	92	50 (54,3%)	42 (45,7%)	0.0281*
N1, N2	11	2 (18,2%)	9 (81,8%)	
Metastatic invasion				
M0	99	50 (50,5%)	49 (49,5%)	1.000
M1	5	2 (40,0%)	3 (60,0%)	
Recurrence (non-invasive cancer only)				
No	36	26 (72,2%)	10 (27,8%)	0.2016
Yes	31	17 (54,8%)	14 (45,2%)	
Progression				
No	63	34 (54,0%)	29 (46,0%)	0.3254
Yes	41	18 (43,9%)	23 (56,1%)	

Fisher’s exact test with 50% lower or higher level of mRNA expression

## Discussion

EMT plays a crucial role in metastasis dissemination and is correlated with poor prognosis in cancer patients [24–26]. However, the precise molecular events that initiate this complex process in bladder cancer are still poorly understood. A mounting body of evidence suggests that dynamic interplay between cancer cells and their microenvironment contributes to metastasis [27–30]. CAFs represent the major component of the TME and have been reported to support tumor progression by a variety of mechanisms [31–35]. However, their role in EMT of bladder cancer cells remains poorly defined.

Thus, we induced the activation of HFs into iCAFs in order to examine their role in bladder cancer cell EMT activation. We found that iCAFs promotes the upregulation of mesenchymal markers, such as N-cadherin and vimentin, while repressing epithelial markers E-cadherin and p-ß-catenin expression in bladder cancer cells. In presence of Wnt ligands, the glycogen synthase kinase 3 beta (GSK3β) is inhibited by phosphorylation, leading to the accumulation and nuclear translocation of β-catenin. In the nucleus, β-catenin binds to the lymphoid-enhancing factor/T-cell factor (LEF/TCF), to initiate the transcription of EMT genes [36–38]. Therefore, the increase expression of phosphorylated GSK3β and decreased expression of the p-β-catenin in RT4 cells cultured in the CM ^iCAF^ suggests a decrease in the β-catenin degradation, confirmed by the total-β-catenin Western blotting. Moreover, EMT transcription factors SNAI1, TWIST1 and ZEB1 were upregulated in the CM ^iCAF^-cultured RT4 cells.

The extent to which specific proteins secreted by CAFs contribute directly to tumor progression is unclear. Previous studies have showed that IL-6 is involved in tumor progression and metastasis in various types of cancer, including lung cancer [39], pancreatic cancer [40], liver cancer [41], gastric cancer [42] and colon cancer [43]. In bladder cancer, clinical findings showed that patients have higher serum IL-6 levels than the healthy controls and that higher IL-6 levels are associated with poorer prognosis [44, 45]. However, the association between CAFs and IL-6 secretion in bladder cancer has not been shown. These studies drove us to investigate the role of IL-6 in bladder cancer. We examined the expression of IL-6 by iCAFs and its receptors on the bladder cancer cell line RT4. We found that IL-6 was upregulated in iCAFs compared to HFs. Similarly, IL-6 have been found to be increased in fibroblasts cultured with the CM from lung cancer [18]. Moreover, RT4 express its receptor IL-6R, indicating that RT4 cells were suitable to respond to the iCAF-secreted IL-6 cytokine. Initiation of metastasis requires EMT, which is characterized by enhanced capability of active locomotion of cancer cells. We found that the iCAF-secreted IL-6 could promote cell growth, migration and invasion significantly. To our knowledge, this is the first study that has shown that iCAF-secreted IL-6 was enough to transform a non-invasive cell line (RT4) into invasive-like cells.

To determine the potential of targeting IL-6 signaling in bladder cancer patients, we performed in silico analysis using *IL-6* expression data acquired from public database. Remarkably, we demonstrated that *IL-6* was expressed at higher levels in bladder cancer stages III/IV than in stages I/II. Moreover, high *IL-6* expression was preferentially associated with high grade MIBC. These results are somewhat consistent with the analysis of Andrews et al., in which a small cohort of human bladder cancer plasma samples revealed IL-6 were associated with cancer stage, metastasis and disease specific survival [44]. Therefore, *IL-6* expression might be related to a more malignant phenotype. In addition, *IL-6* correlates with CAF marker *ACTA2* and negatively correlates with tumor purity, suggesting that IL-6 is produced primarily by CAFs and not tumor cells. Nonetheless, our analysis revealed a correlation between high *IL6/ATCA2* mRNA levels and poor cancer specific survival.

Taken together, our results reveal that IL-6 secreted from iCAFs promotes malignant behavior through the activation of the EMT program in bladder cancer cells. These results provide a mechanistic explanation for the role of IL-6 in the bladder cancer microenvironment, as well as the correlation observed between high IL-6 levels and metastatic potential in bladder cancer patients. Overall, our findings may serve as an attractive therapeutic target for human bladder cancer driven by IL-6 signaling. In this study, we demonstrated that IL-6 blockade reverses the effect of CAFs on tumor progression. Therefore, since IL-6 blocking antibodies are already approved by the Food and Drug Administration (FDA), such an approach to impede CAFs functions may be a clinical promising strategy. In solid tumour model, siltuximab, an IL-6 antibody, has demonstrated antitumor efficacy against ovarian, prostate, and lung cancers [16]. Despite these preclinical data, there remains a dearth of clinical trials investigating targeted approaches, such as *IL-6* signaling inhibition, particularly in bladder cancer. IL-6 signal through multimeric complexes that includes the gp130 receptor/IL-6Rα chain and ultimately triggers a signaling cascade that is mediated by STAT3 pathway. STAT3 activation was demonstrated to drive the proliferation, survival, invasiveness, and metastasis of tumor cells, while strongly suppressing the antitumour immune response [16]. Moreover, STAT3 activation was associated with bladder cancer cell growth and survival [46]. In our study, we observed that iCAFs activates the STAT3 signaling pathway in RT4 cells. OPB-31121 and OPB-51602 are orally administrated STAT3 inhibitors, which are capable of binding to the SH2 domain of STAT3, and disrupt STAT3 dimerization and DNA binding activity, and are currently under evaluation in clinical trials (NCT00657176, NCT01406574, NCT01423903 and NCT01184807). A combination of these two drugs could be used to more efficiently target some of the downstream mediators of IL-6 signaling.

Our study has some limitations. The TME consists of cancer cells, fibroblasts, endothelial cells, and immune cells, all of which contribute to the tumor secretome. Although our findings were focused on the interaction between tumor cells and CAFs, IL-6 is a multifunctional cytokine known to be secreted by and to influence multiple cell types in the TME, from immune to endothelial cells, and therefore, it is important to keep in mind the potential influence of these cells in our system [19, 47]. Thus, this strongly indicates that neutralization of IL-6 signaling could have even more potent antitumor effects.

## Conclusion

Identify factors involved in the dynamic interaction between tumor cells TME is much-needed to improve cancer therapy. Herein, we show how one of the mediators of such an interaction, namely IL-6, mainly secreted by CAFs, can support tumor progression and how it can be antagonized by a neutralizing antibody to its receptor, which significantly reduces proliferation, migration and invasion in bladder cancer cells. These results highlight a prominent role for CAFs in bladder cancer and provide a framework for further studies to develop relevant TME-based anti-cancer therapy.

## Additional files

Additional file 1: Exosomes characterization. **A.** TEM micrographs showing morphology of exosomes immunoprecipitated with anti-CD9 mAb from bladder cancer cells. Exosomes were stained with 2% uracyl acetate after being placed on carbon-coated TEM grid. **B.** NanoSight analysis show three repeated measures of exosomes isolate according to their size. (PDF 945 kb)

Additional file 2: Correlation of tumor purity scores obtained by using the ABSOLUTE algorithm with mean *IL6* (**A**) and *ACTA2* (**B**) expression. (PDF 163 kb)

## Abbreviations

CAF: Cancer-associated fibroblast
CM: Conditionned medium
DMEM: Dulbecco-Vogt modified Eagle’s media
EMT: Epithelial-mesenchymal transition
FAP: Fibroblast associated protein
FBS: Fetal bovine serum
FDA: Food and Drug Administration
GSK3β: Glycogen synthase kinase 3 beta
HF: Healthy fibroblast
iCAF: Induced cancer-associated fibroblast
IL-6: Interleukin 6
IL-6R: IL-6 receptor
LEF: Lymphoid-enhancing factor
MIBC: Muscle-invasive bladder cancer
NMIBC: Non-muscle-invasive bladder cancer
pSTAT3: Phosphorylated STAT3
STAT3: Signal transducer and activator of transcription 3
TCF: T-cell factor
TME: Tumor microenvironment
α-SMA: Alpha smooth muscle actin
