# Supplementary material for: Cancer-associated fibroblasts induce epithelial–mesenchymal transition of bladder cancer cells through paracrine IL-6 signalling
Source: BMC Cancer. 2019 Feb 11;19:137. doi: 10.1186/s12885-019-5353-6 (PMC6371428; doi:10.1186/s12885-019-5353-6)
Supplement: Supplementary file 1 — Exosomes characterization. A. TEM micrographs showing morphology of exosomes immunoprecipitated with anti-CD9 mAb from bladder cancer cells. Exosomes were stained with 2% uracyl acetate after being placed on carbon-coated TEM grid. B. NanoSight analysis show three repeated measures of exosomes isolate according to their size. (PDF 945 kb) [file 12885_2019_5353_MOESM1_ESM.pdf]

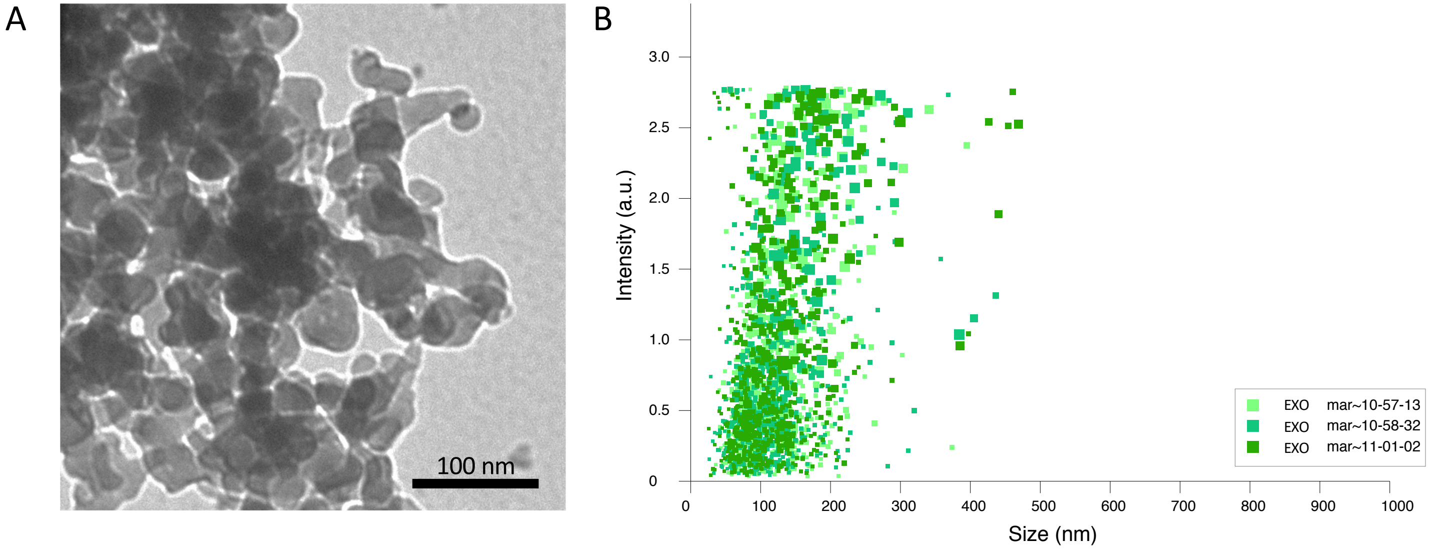

Additional file 1. Exosomes characterization. A. TEM micrographs showing morphology of exosomes immunoprecipitated with anti-CD9 mAb from bladder cancer cells. Exosomes were stained with 2% uracyl acetate after being placed on carbon-coated TEM grid. **B.** NanoSight analysis show three repeated measures of exosomes isolate according to their size.
