# Supplementary material for: Cancer-associated fibroblasts induce epithelial–mesenchymal transition of bladder cancer cells through paracrine IL-6 signalling
Source: BMC Cancer. 2019 Feb 11;19:137. doi: 10.1186/s12885-019-5353-6 (PMC6371428; doi:10.1186/s12885-019-5353-6)
Supplement: Supplementary file 2 — Correlation of tumor purity scores obtained by using the ABSOLUTE algorithm with mean IL6 (A) and ACTA2 (B) expression. (PDF 163 kb) [file 12885_2019_5353_MOESM2_ESM.pdf]

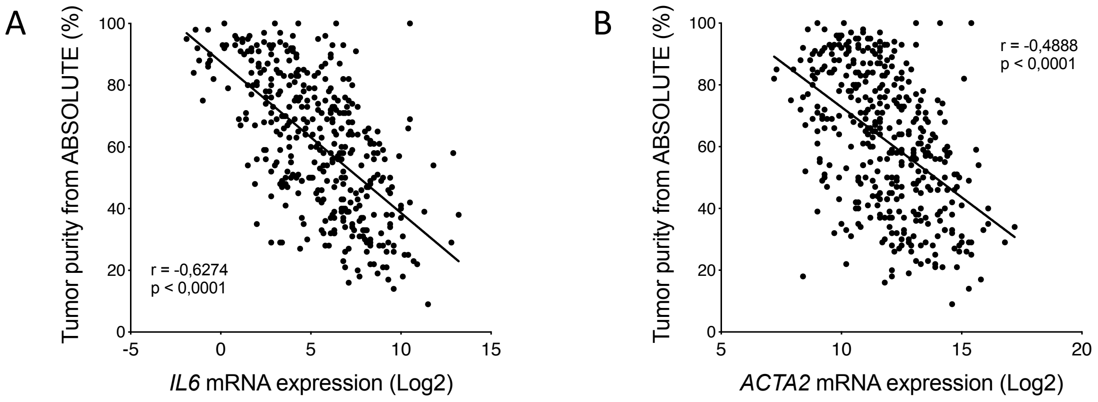

Additional file 2. Correlation of tumor purity scores obtained by using the ABSOLUTE algorithm with mean *IL6* (**A**) and *ACTA2* (**B**) expression.
